# Treatment of peri-implant mucositis using an Er:YAG laser or an ultrasonic device: a randomized, controlled clinical trial

**DOI:** 10.1186/s40729-025-00591-0

**Published:** 2025-01-24

**Authors:** Viveca Wallin Bengtsson, Akira Aoki, Koji Mizutani, Christel Lindahl, Stefan Renvert

**Affiliations:** 1 Department of Oral Health, Faculty of Oral Health Science, Kristianstad University, 291 88 Kristianstad, Sweden; 2 Department of Periodontology, Graduate School of Medical and Dental Sciences, Institute of Science Tokyo, Yushima, Bunkyo-Ku, Tokyo, Japan; 3 Blekinge Institute of Technology, Karlskrona, Sweden; 4 Division of Restorative Dentistry and Periodontology, Dublin Dental University Hospital, Trinity College Dublin, Dublin, Ireland

**Keywords:** Er:YAG laser, Patient-reported outcome measures, Peri-implant mucositis, Professional mechanical plaque removal, Ultrasonic scaler

## Abstract

**Purpose:**

The study assessed the clinical outcomes following treatment of peri-implant mucositis using Er:YAG laser or an ultrasonic device over six months. Patients' experience of pain, aesthetics, and Quality of life were further assessed.

**Methods:**

One dental implant, per included patient, diagnosed with peri-implant mucositis underwent treatment with an Er:YAG laser (test) or an ultrasonic scaler (control) randomly. Treatments were performed at baseline and months three and six. At each session, oral hygiene was instructed after plaque registration, and the patient was guided in proper cleaning technique using a toothbrush and interproximal aids as needed. Full mouth bleeding on probing (FMBoP), full mouth plaque score (FMPS), implant bleeding on probing (BoP), implant mean graded bleeding (mBI), implant probing pocket depts (PPD), implant suppuration and bone levels were assessed. Oral health-related Quality of life (OHQoL) and visual analog scales (VAS), which reflect aesthetic satisfaction and pain of the treatment, were also evaluated.

**Results:**

Forty-six patients were included. FMBoP was significantly reduced from 30.1 to 21.5% (test) (p < 0.001) respectively from 35.0% to 30% (control) (p < 0.01). FMPS showed significant reduction from 61.5 to 32.7% (test) (p < 0.001) and from 58.7 to 39.1% (control) (p < 0.001). At the implant BoP reduced from 89.0 to 55.7% (test) (p < 0.001) respectively from 94.9 to 63.7% (control) (p < 0.001). mBI was reduced from 1.3 to 0.6 (test) (p < 0.01) and from 1.9 to 0.8 (control) (p < 0.001). Distribution of “no bleeding” increased from 13 to 61% (test) (p < 0.05) and from 0 to 35% (control) (p < 0.05). At month three, statistically significant intergroup differences were shown for PPD ≥ 4 mm with 43.5% (test) respectively 73.9% (control) (p < 0.05). At month six, statistically significant intergroup differences, were shown for FMBoP 21.5% (test) respectively 30% (control) (p < 0.05) and for plaque score at the implant 4.0% (test) respectively 26% (control) (p < 0.05). Less pain was reported in the laser group at three days 0.08 (test) respectively 0.2 (control) (p < 0.05).

**Conclusions:**

Treatment of peri-implant mucositis was effective regardless of whether the treatment was performed with an Er:YAG laser or an ultrasonic scaler. Fewer diseased sites were diagnosed at six months following laser treatment.

*Trial registration*: Registered at www.clinicaltrials.gov: study no, NCT05772299.

## Introduction

Teeth that have been lost are often replaced with dental implants. The prognosis for dental implant treatment is good, and a survival rate of 93% has been reported after 20 years [1]. However, peri-implant mucositis or peri-implantitis is a common complication. On a patient level, the prevalence of peri-implant mucositis ranges from 1 to 47% [2], and meta-analyses estimated a weighted mean prevalence of peri-implant mucositis of 43% (CI: 32–54%) [3].

The objective of peri-implant mucositis treatment is to eliminate or significantly reduce the levels of pathogens to a level resulting in a clinically healthy situation [4, 5] and the therapy should be evaluated 2–3 months after treatment [6].

Dental implants generally have a screw-shaped design, and to facilitate healing and osseointegration, the surface structure is often rough. Treatment models effectively used to treat the tooth's root surfaces with periodontal diseases, like submarginal instrumentation, are challenging to use on the rough threaded implant surface [5, 7, 8].

The European Federation of Periodontology (EFP) clinical practical guideline 2023 recommends self-performed, effective oral hygiene and professional mechanical plaque removal (PMPR) when treating peri-implant mucositis [6]. PMPR aims to reduce soft tissue inflammation by removing hard and soft deposits from the surface of the dental implant and the supra-structure. PMPR can be performed using an ultrasonic device with plastic or carbon-coated tips, an air-polishing device, plastic, carbon or titanium curettes, and lasers [6].

Randomized clinical trials evaluating laser treatment of peri-implant mucositis are lacking [3]. Patient-reported outcome measures (PROMs) have only infrequently been studied evaluating the prevention and management of peri-implant mucositis and peri-implantitis [3, 9] and especially not postoperative pain, aesthetics related to the treatment and the effect on Quality of life (OHRQoL) [10]. The aim of the present study was to assess clinical outcomes following treatment of peri-implant mucositis using either an Er:YAG laser device or an ultrasonic device over six months. A second aim was to assess patients' experience of pain, aesthetics, and Quality of life in treating peri-implant mucositis.

## Methods

### Study design

This study was a single-blinded randomized six-month clinical trial (RCT) approved by the National Ethics Review Board in Uppsala, Sweden (2020-02586). The RCT was conducted according to the World Medical Association Declaration of Helsinki principles, revised in 2013. The CONSORT guidelines for clinical trials were followed. The study was registered under www.clinicaltrials.gov (study no. NCT05772299).

### Study population

The patients were referred for a treatment of peri-implant mucositis. Sixty-four patients were screened and reviewed for medical history and available radiographs. Potential cases, according to the inclusion and exclusion criteria, further underwent a full mouth routine periodontal examination. Forty-six subjects who fulfilled the inclusion criteria and consented were consecutively included.

### Inclusion criteria—presence of peri-implant mucositis

- One dental implant with a minimum of one or more peri-implant sites with probing depth ≥ 4 mm combined with bleeding and/or pus on probing
- A limited allowed bone loss ≤ 2 mm measured from the implant shoulder (changes resulting from initial bone remodelling)

### Exclusion criteria

- Subjects with uncontrolled diabetes mellitus (HbA1c > 6.5)
- Subjects requiring prophylactic antibiotics
- Subjects taking prednisolone
- Subjects taking medications with effects on gingival growth
- Subjects taking systemic antibiotics in the preceding month

For confidentiality, coding was used and saved according to the regulations of Kristianstad University, Sweden. The patients were referred for treatment between 2021 and 2023. Due to Covid 19, when no patient treatment was performed the start of the study was delayed. Before entering the study, a preparatory treatment phase was performed, including instruction in oral hygiene measures and debridement of teeth and implants not to be part of the study. Good patient motivation and compliance were also identified. The patients were informed about the study and filled out written consent.

### Randomization

After entering the study, patients were randomly assigned to treatment with an Er:YAG laser (test) or an ultrasonic device (control) using pre-prepared randomization according to pre-defined computerized randomization.

### Measurements

The clinical study was conducted at the speciality clinic of periodontology at Kristianstad University. Unaware of the treatment, the same examiner (CLL) performed the measurements, and the patients were asked not to talk about their treatment. Measurements were recorded at baseline and in months one, three, and six using a manual plastic probe tip diameter of 0.5 mm (*Colorvue* Probe, *Hu-Friedy*, *USA*) and probing force of 0.2 N., The following measurements were recorded for the full mouth: bleeding on probing (FMBoP) (4 sites/tooth, 6 sites/implant) [11], plaque score (FMPS) (4 sites/tooth, 6 sites/implant) [12]. Measurements at the implant were: bleeding on probing (BoP) (6 sites), severity of bleeding graded 0–3 0 = no bleeding, 1 = a dot of bleeding, 2 = a line of bleeding, and 3 = a drop of bleeding (6 sites) (i.e. modified bleeding index or mBI) [5, 13], suppuration (6 sites), plaque (6 sites), probing pocket depths (PPDs) (6 sites), hyperplasia, buccal recession of the mucosal margin.

Patient evaluation of the treatment: Oral health–related quality of life (OHQoL) was measured with the Oral Health Impact Profile–14 version (OHIP-14) [14] at baseline and month six. The Quality of life was expressed as the sum of the fourteen questions, a maximum of 56 scores. The mean values were calculated. A lower score indicates high OHQoL, and a higher score indicates low OHQoL. The analysis did not include patients who had not answered all questions.

Visual Analogue Scales consisted of a 10-point graded straight horizontal line, with the left end indicating 0 "no pain and the right end indicating 10 "worst pain". The VAS concerned pain during treatment, the same day, day two and day three after the treatment. Further, the other VAS reflected the patients' aesthetic satisfaction at baseline and month six. The left end on the VAS indicated "not at all satisfied", and the right was "very satisfied". Each patient's mean visual analogue score was calculated.

### Radiographic examination

Intraoral digital periapical standardized radiographs of the site of interest were taken with a bite-block to detect loss of bone at baseline and month six. Radiographs were analysed by an independent and blinded examiner (VWB) using the Synedra View software for personal computers. The known distance between implant thread pitch lengths was used to calibrate the images. The distance between the implant shoulder and the most superior alveolar bone level on the mesial and distal aspects of the implant was measured and merged. The intra-class coefficient (ICC) for the distance between the implant shoulder and the alveolar bone level was 0.94 (95% CI: 0.84–0.98, p < 0.00).

### Treatment procedures

**Fig. 1:**
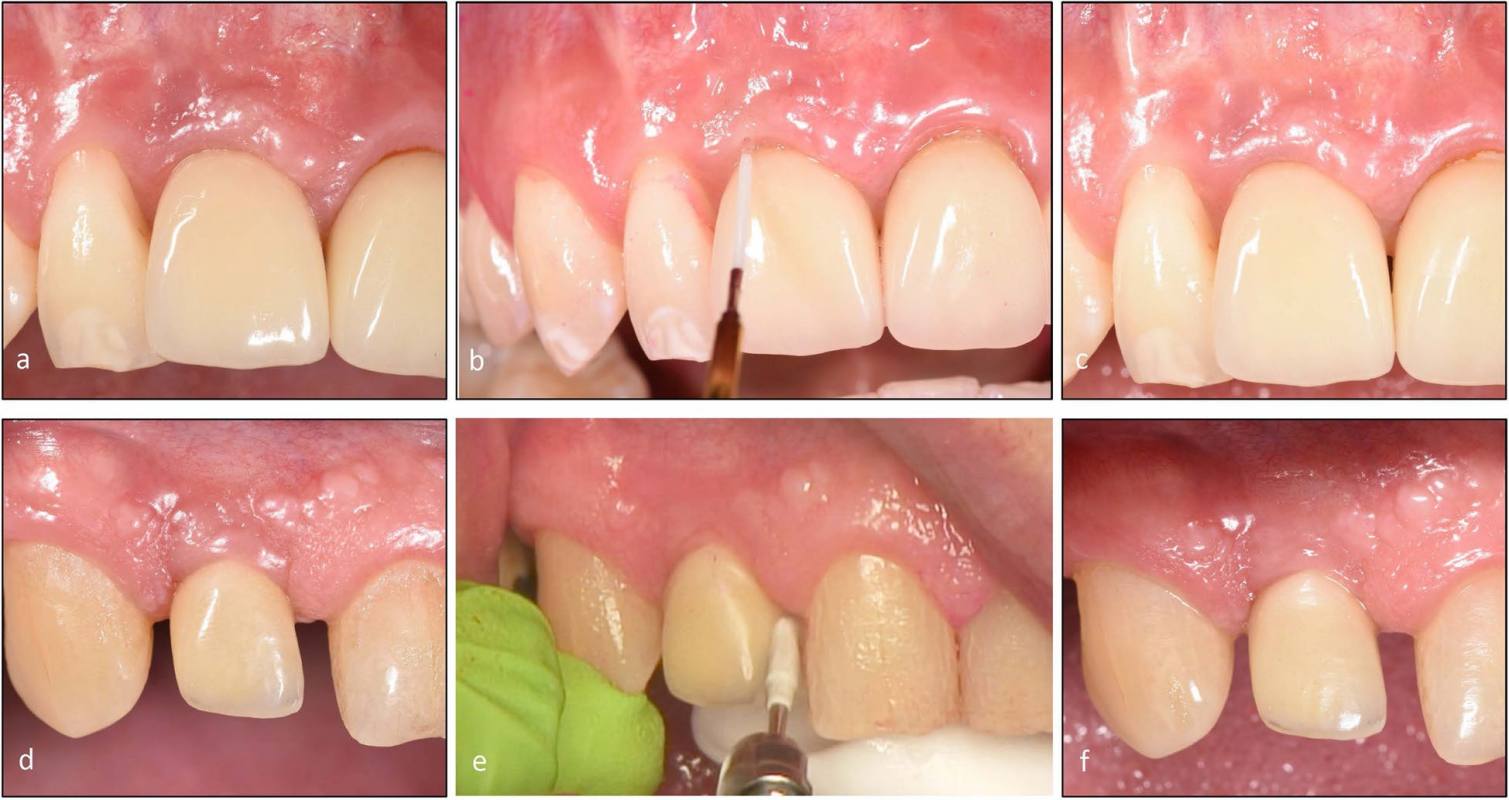
Peri-implant mucositis before, during and after treatment with Er: YAG laser or ultrasonic scaler. **a** Peri-implant mucositis 11 before treatment **b** Er: YAG laser treatment 11 **c** six months after Er: YAG laser treatment 11 **d** Peri-implant mucositis 12 before treatment **e** ultrasonic scaling 12 **f** six months after ultrasonic scaling 12

The same treatments were performed at baseline, months three and six. Final evaluation data was provided in month six. All treatments were performed by a non-blinded periodontist (VWN). No implant-supported constructions were removed, and no local anaesthesia was used. At each session and after plaque registration, the patients were instructed to use proper home care using a toothbrush and interproximal aids as needed. Treatment in the test group (test) was done using an Er: YAG laser (Erbium-doped yttrium–aluminium-garnet; Er: YAG, AdvErL EVO, Morita Corporation, Japan—> J. MORITA MFG. CORP., Japan) with tip PS400TS and power 50 mJ, frequency 10 PPS and proportion air/water 7/7 [15]. The tip was placed in the peri-implant pocket, and a moving motion was from bottom to coronal at the implant surface, with a sweeping motion covering the soft tissue. The treatment in the control group (control) was performed with an ultrasonic device (Electro Medical Systems, EMS) with a plastic-coated tip (PI Instrument Nyon; EMS, Nyon, Switzerland). The tip was placed in the peri-implant pocket and used in a similar way as described above (Fig. 1). All treatments were finalized by polishing with a rubber cup and polishing paste. Postoperative complications were asked for in the examination protocol as an open question at each examination, and treatment time was registered.

At month one, measurements, oral hygiene instructions, and polishing with a rubber cup and polishing paste were performed (CLL).

### Statistical analysis

The IBM SPSS version 27.0 statistical software package (SPSS Inc., Armonk, NY, USA) for personal computers was used in the analyses. The statistical unit was based on both the patient and the implant. Descriptive and comparative statistics were used when analysing the data. The primary outcome measure in this study was bleeding on probing (BoP). A power analysis was performed, and thirty-eight patients were minimum to reach a clinically significant difference at ± 30% reduction in bleeding of probing (BoP) between the groups with alpha 0.05 and power 80%. Forty-six individuals were included in the study, and all analyses were based on the "per protocol" principle. Mean values, standard deviation (SD) and frequency distributions are given. Independent t-test (equal variance not assumed) and paired t-test were used to compare inter- and intra-group differences. The chi-square test and non-parametric McNemar test were used for categorical variables. Statistical significance was set at p < 0.05.

## Results

**Fig. 2:**
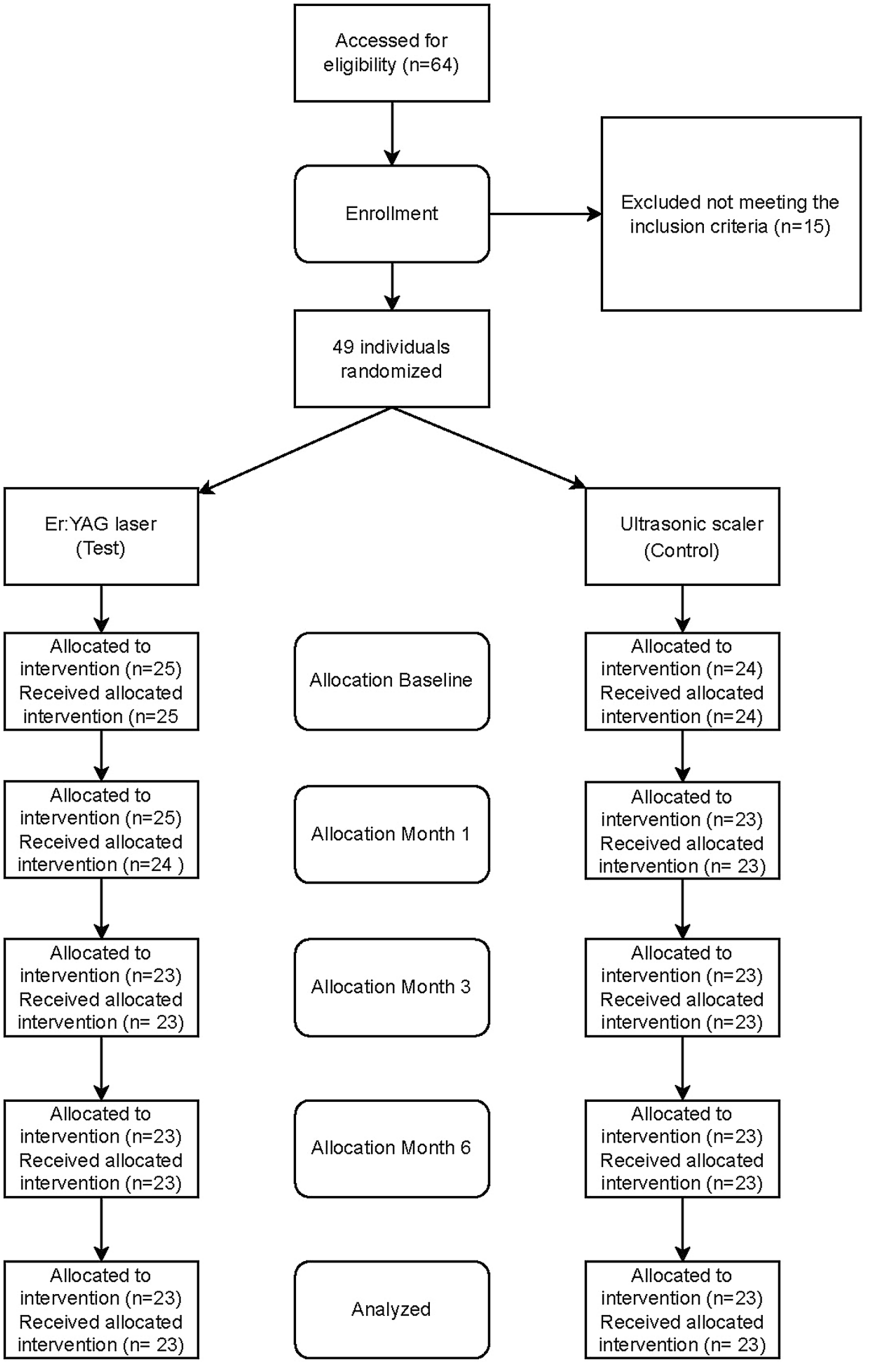
Consort flow chart

This study was based on 46 patients (56.5% women) treated per protocol (Fig. 2). The mean age was 64.4 years ± 13.8 range 29–82. For baseline characteristics, see Table 1.

**Table 1:** Baseline characteristics of the patients in percent and for packyears mean ± SD

Variables	Test group %	Control group %	Total patients %
Cardiovascular diseases	43.5%	47.8%	45.7%
Diabetes mellitus	8.7%	8.7%	8.7%
Current smokers	4.3%	8.7%	6.5%
Packyears in current smokers (years)	36 ± ^†^	6 ± 1.4	16 ± 17.3
Former smokers	34.8%	47.8%	41.3%
Using prescribed medicines	47.8%	65.2%	56.5%

Test group = Er:YAG laser, control group = Ultrasonic scaler

^†^NA = not applicable

After treatment at baseline, postoperative complications were reported by 4/46 (8.9%), including irritated mucosa (test), swollen mucosa (test), pain when touching (test), and a feeling of pressure (control). At month three, postoperative complications were reported by two individuals. One from the test group reporting bleeding, and one from the control group reported soreness in the corner of the mouth.

Of the treated implants, 50.0% were Astra Tech, 37.0% were Nobel BioCare, 2.2% were Biomet 3i, and 4.3% were unidentified. Treatment time was for the test: 3.5 min at baseline, 2.8 min at month three and 2.9 min at month six. The corresponding figures for the control were 3.8 min at baseline, 3.1 min at month three and 2.7 min at month six. No significant intergroup differences existed.

### Full mouth clinical indices

At month three, FMBoP in the test was 20.8% ± 8.9, and in the control, 29.8% ± 17.2 (CI: − 17.3 to − 0.8; p = 0.03). Intergroup difference was also shown at month six, with FMBoP in the test 21.5% ± 9.0 and 30.0% ± 15.7 in the control (CI: − 16.1 to − 0.8; p = 0.03). FMBoP was significantly reduced in both groups (Table 2).

**Table 2:**
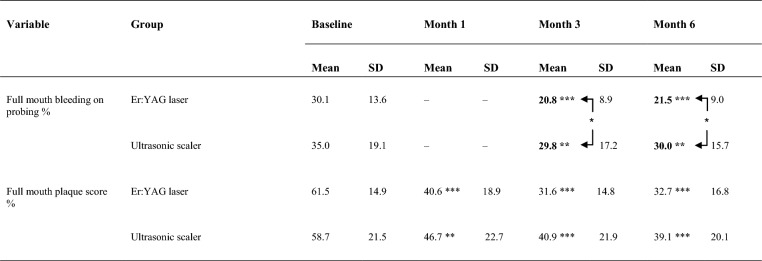
Full-mouth bleeding on probing and plaque scores at different timepoints mean ± SD

Significant intergroup differences at months three and six in full mouth bleeding on probing

*p < 0.05 statistically significant difference in respective group at different timepoints compared to baseline. ** p < 0.01 statistically significant difference in respective group at different timepoints compared to baseline. *** p < 0.001 statistically significant difference in respective group at different timepoints compared to baseline

Arrows in the hook clamps show statistically significant difference * p < 0.05 between groups at month three and six

Full mouth plaque score was significantly reduced in both groups compared to baseline. No statistical intergroup differences existed (Table 2).

### Implant clinical indices

#### Bleeding on probing

In the test, BoP was reduced from 89.0% ± 13.9 to 55.7% ± 22.8 (CI: 22.9–43.7, p = 0.00), and in the control, from 94.9% ± 10.6 to 63.7% ± 21.6 (CI: 23.0–39.4; p = 0.00) (Table 3). No statistical intergroup differences existed in BoP.

**Table 3:**
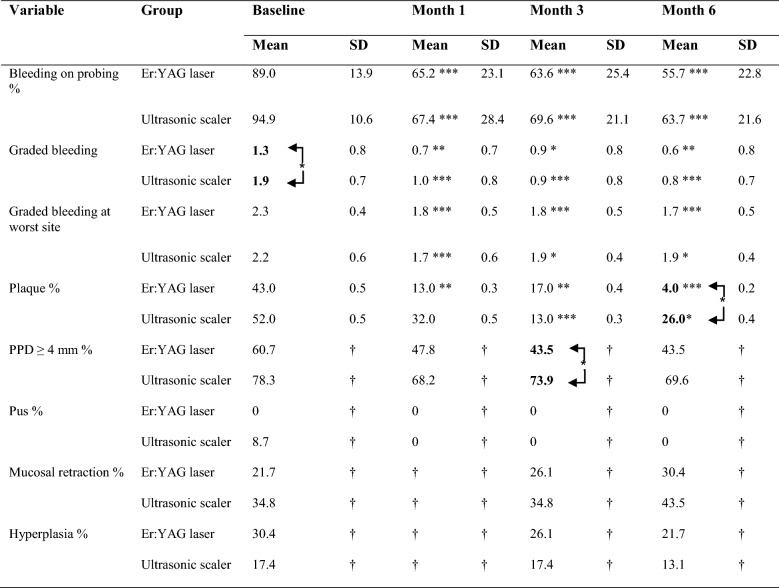
Implant bleeding, scores (mean ± SD) and prevalence of implant probing pocket depths ≥ 4 mm, pus, and mucosal changes (%) at different timepoints on six sites

Significant intergroup differences at baseline in graded bleeding, at month three in probing pocket depth (PPD) ≥ 4 mm and at month six in plaque

*p < 0.05 statistically significant difference in respective group at different timepoints compared to baseline. ** p < 0.01 statistically significant difference in respective group at different timepoints compared to baseline. *** p < 0.001 statistically significant difference in respective group at different timepoints compared to baseline

Arrows in the hook clamps show statistically significant difference * p < 0.05 between groups. † NA = Not applicable

#### Severity of bleeding

**Fig. 3:**
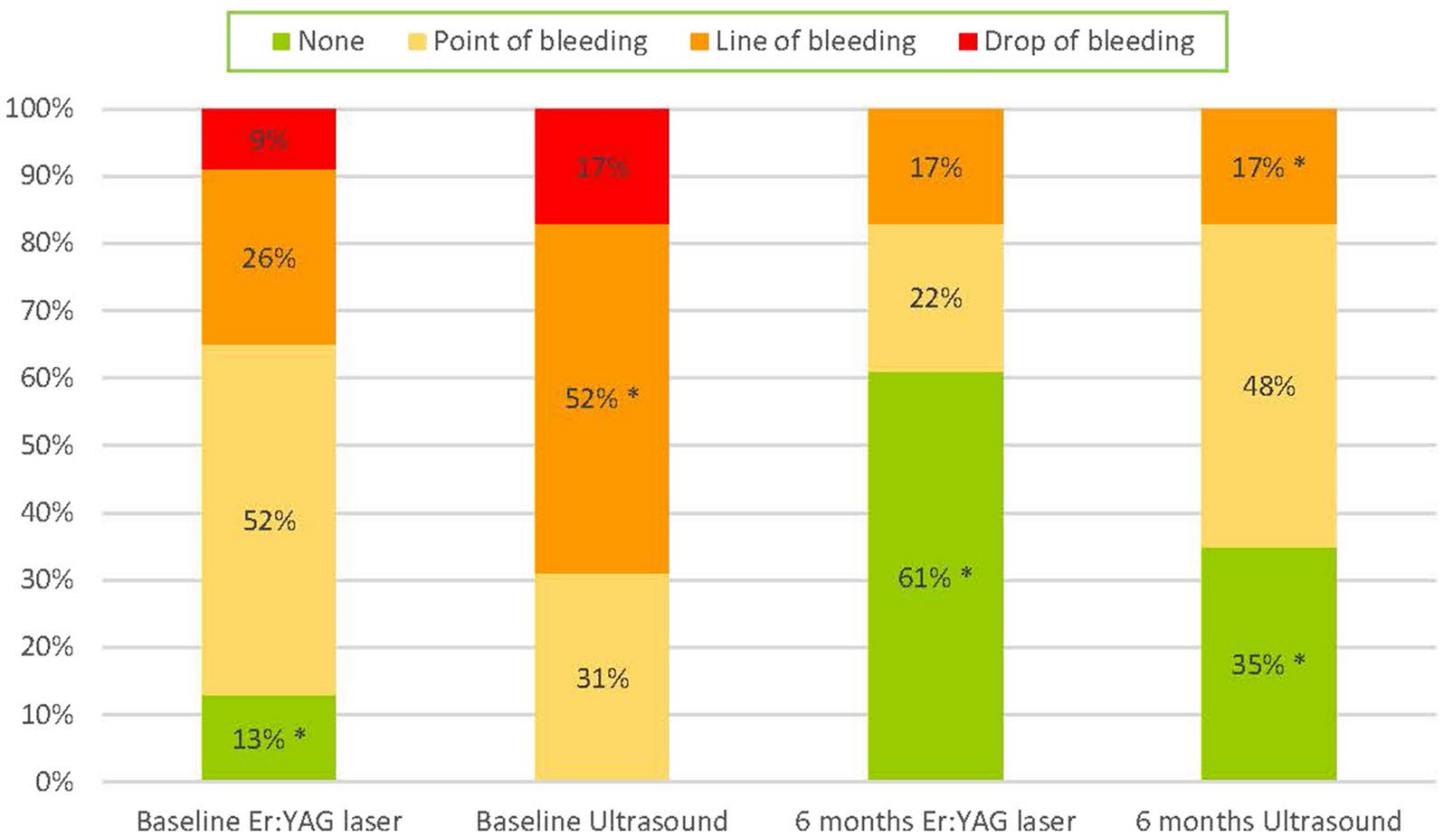
Distribution of graded bleeding on probing i.e. the severity at the implant surfaces (6 surfaces per implant) in both groups at baseline and six months. *p < 0.05 statistically significant difference from baseline to six months in respective group

The mBI was significantly reduced in both groups (Table 3). No intergroup differences in mean graded bleeding (mBI) were shown. For the distribution of bleeding, see Fig. 3.

#### Presence of plaque

Plaque was significantly reduced from baseline to 6 months from 43.0% ± 0.5 to 4.0% ± 0.2 in the test (CI: 0.2–0.6; p = 0.00) and from 52.0% ± 0.5 to 26.0% ± 0.4 in the control (CI: − 0.0–0.5; p = 0.03). At month six the presence of plaque was 4.0% ± 0.2 in the test and 26.0% ± 0.5 in the control (CI: − 0.4 to − 0.0; p = 0.04) (Table 3).

#### Peri-implant probing pocket depth (PPD)

At baseline, the mean PPD was in the test, 4.0 mm ± 1.3 and in the control, 4.8 mm ± 1.2 (CI: − 1.6 to − 0.8; p = 0.03). At month six, the mean PPD in the test was 3.6 mm ± 1.4, and in the control, the mean PPD was 4.3 mm ± 1.1 (CI: 0.1–0.7; p = 0.05). In both groups, the reduction in mean PPD was statistically significant in the test (CI: 0.1–0.7; p = 0.02), and in the control (CI: 0.2–0.7; p = 0.00).

As a result of therapy, the prevalence of PPD ≥ 4 mm decreased. At month three, the prevalence of PPD ≥ 4 mm was 43.5% in the test and 73.9% in the control (CI: 1.1–12-8; p = 0.03). The reduction in prevalence of PPD ≥ 4 mm from baseline to months three and six was for the test 17.2%, and in the control, the reduction at month three was 4.4% and at month six 8.7%.

At month six, 26% of the implants in the test demonstrated sites with a PPD ≥ 4 mm + BoP and or pus, respectively 52% in the control (CI: 0.9–10.7; p = 0.07).

### Radiographic images

At baseline radiographs, the mean distance of the merged mesial and distal surfaces of the study implant was 0.9 ± 0.83 mm in the test and 1.1 ± 0.62 mm in the control (CI: − 0.7 to 0.2; p = 0.32). At month six, the corresponding figures were 0.9 ± 0.80 mm and 1.1 ± 0.58 mm in the test, respectively, in the control (CI: − 0.6 to 0.2; p = 0.40). Intragroup comparisons showed no statistical differences.

### Patient-reported outcome measures

#### Pain

Pain during treatment reported on a VAS scale was at baseline 2.6 ± 2.6 in the test and 3.9 ± 3.0 in the control (CI: − 3.0 to 0.5; p = 0.15) and three days posttreatment 0.08 ± 0.1 in the test and in the control 0.2 ± 0.3 (CI: − 0.3 to − 0.0; p = 0.04) (Table 4).

**Table 4:**
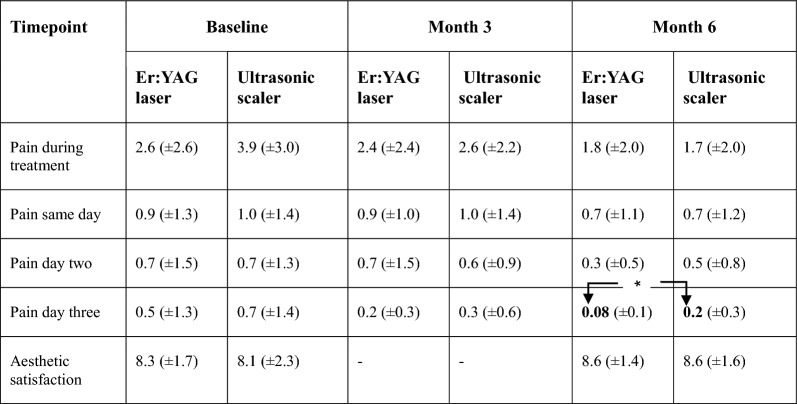
Patient-reported outcomes on visual analogue scales of treating peri-implant mucositis with Er:YAG laser or ultrasonic scaler mean ± SD

Significant intergroup differences at month six in pain day three

Arrows in the hook clamp show a statistically significant difference *p < 0.05 between groups at month six. For the pain visual analogue scale, 0 indicates “no pain”, and 10 indicates “worst pain”. For aesthetics the visual analogue scale 0, indicating “not at all satisfied”, and 10, indicating “very satisfied”

#### Aesthetics

Satisfaction with the aesthetics was in the test 8.3 ± 1.7 at baseline, and 8.1 ± 2.3 in the control (CI: − 1.0 to 1.4; p = 0.69). At month six it was 8.6 ± 1.4 in the test and 8.6 ± 1.6 in the control (CI: − 0.9 to 0.9; p = 0.92) (Table 4).

#### Quality of life

At baseline, the total OHIP-14 score in the test was 1.27 ± 1.9, and 4.26 ± 5.3 in the control (CI: − 5.4 to − 0.6; p = 0.00). The total OHIP-14 score at month six in the test was 1.48 ± 2.5, in the control, 2.17 ± 3.4 (CI: − 2.5 to 1.1; p = 0.22). No statistical differences over time were observed in any of the groups.

## Discussion

The present study assessed, the treatment of peri-implant mucositis using either an Er:YAG laser or an ultrasonic device. FMBoP and FMPs were significantly reduced in both groups. At the implant level, significant reductions were shown in the extent and severity of bleeding for both groups.

A few clinical randomized studies on peri-implant mucositis treatment evaluating a single mode of treatment are reported [16, 17]. Er:YAG laser has been used in another randomized clinical trial to treat peri-implant mucositis [18]. In that study, the Er:YAG laser was used in combination with titanium curettes, and the effectiveness of the Er:YAG laser could, therefore, not be evaluated.

The primary outcome measure in the present study was change in BoP. The reduction of BoP at the implant in the Er:YAG laser group was 33.3%, compared to 31.2% in the ultrasonic group. In another randomized clinical study, BoP at the implant was reduced by 27.2% at implants treated by a glycine powder air-polishing device compared to 30.5% at implants treated by the same ultrasonic scaler used in the present study [17]. The reduction of BoP in the two studies is similar. In the study by Riben-Grundstrom et al. [17], the BoP figures are, however, lower than those in the current study. The higher baseline implant bleeding values may have negatively affected the final treatment results in the present study. It has been demonstrated that BoP ˃50% of the implant sites predict disease progression [19, 20].

In this study, mBI was significantly reduced over time in both groups. In the Er:YAG laser group, mBI reduced from 1.3 at baseline to 0.6 at month six, and the figures for the ultrasonic group were from 1.9 to 0.8. These reductions in mBI are equivalent to two previous studies demonstrating reductions in mBI of 0.6–0.8 at six months [16, 21]. One of them compared combined ultrasonic scaling and glycine powder air-polishing with ultrasonic scaling alone [21], and the other study compared a chitosan rotating brush as a single mode with titanium curettes [16]. The importance of mBI in predicting progressive bone loss remains questionable [10]. Maybe the severity of bleeding, using the grading of bleeding [13], is a better indicator of treatment needs than just measuring BoP. The EFP clinical practice guideline 2023 involving peri-implant mucositis highlights the importance of profuse bleeding at implants [6]. Re-treatment is recommended if two or more surfaces at the implant demonstrate profuse bleeding [6]. Possibly bleeding grade 1, "a point of bleeding", and even bleeding grade 2 ", a line of bleeding", may constitute the consequence of traumatizing the mucosa while measuring the pocket and may not represent an inflammatory response. If so, this may risk falsely raising the extent of bleeding [22]. Using this way of reasoning and adding bleeding grade 1 to grade 0, at month six, in the Er:YAG laser group, 83.0% of the surfaces and 83.0% in the ultrasonic group did not demonstrate bleeding due to inflammation. Wohlfahrt et al. [16] also measured the severity of bleeding with the same grading system as in the present study. When grade 1 and grade 0 were combined, the authors reported that 86.0% of the surfaces in the chitosan group and 79.0% in the titan curette group were bleeding-free after treatment. These figures represent the severity of bleeding when considering bleeding grade 1 as traumatic and not inflammatory-driven bleeding and are equivalent to what is reported in the present study. If grade 1 to grade 0 were added concerning the severity of bleeding, ten implants would have been considered successfully treated (seven from the Er:YAG laser group and three from the ultrasonic group).

An increase in PPD is often a sign of peri-implant mucositis [23], therefore, a reduction could be considered a sign of healing. Following treatment, there was a reduction in the prevalence of PPD ≥ 4 mm in both groups. At month three the prevalence of probing pocket depths ≥ 4 mm in the Er:YAG laser group was 43.5% and significantly lower compared with the ultrasonic group, 73.9% (p = 0.03). The reduction of pockets observed at month three remained in the test group until month six. In the control group, there was some further improvement.

PROMs are seldom described in studies following the treatment of peri-implant mucositis [3]. In the present study, PROMS were included as outcome variables. Pain experienced during treatment decreased over time and was lower than reported by Wohlfhart et al. [16]. The studies used different modes of treatment and are, therefore, difficult to compare. It could, however, be noted that the pain sensation was low regardless of the therapy used in the present study. Three days after treatment at month six, Er:YAG laser group patients reported a statistically lower pain sensation than those in the ultrasonic group. In a meta-analysis by Mikami et al. [24] reporting pain following non-surgical periodontal treatment with either an Er:YAG laser or conventional root planning, significantly lower patient-reported pain was reported in the laser group.

A limitation of the present study is the difference in mean PPD at baseline between groups. The shallower PPD in the test group may have limited the healing potential following therapy and, thus, the potential intergroup differences. Another limitation of the present study is that the level of the sub- or supra-mucosal restoration margin was not registered. The placement of the restoration margin may have influenced the treatment results [25]. It has been demonstrated that the width of the keratinized mucosa may affect the treatment outcomes [18, 26, 27]. A limitation of the present study is that the width of the keratinized mucosa was not measured.

The EFP clinical practice guidelines 2023 recommend regular supportive care in patients with healthy peri-implant tissues to prevent the development of peri-implant diseases [6]. If peri-implant mucositis has developed, self-performed, effective oral hygiene, and professional mechanical plaque removal, are recommended [6]. Management of peri-implant mucositis is considered a preventive measure for the onset of peri-implantitis and must be treated [6]. This study demonstrated that treating peri-implant mucositis using an Er:YAG laser or an ultrasonic scaler is effective, and both therapies can therefore be recommended for treating peri-implant mucositis.

## Conclusions

Er:YAG laser and ultrasonic scaler as single modes of therapy were effective in treating peri-implant mucositis and reduced bleeding and probing pocket depths. Fewer diseased sites were diagnosed at six months following laser treatment.

## Data Availability

No datasets were generated or analysed during the current study.
