# Correction: Treatment of peri-implant mucositis using an Er:YAG laser or an ultrasonic device: a randomized, controlled clinical trial

**DOI:** 10.1186/s40729-025-00599-6

**Published:** 2025-02-26

**Authors:** Viveca Wallin Bengtsson, Akira Aoki, Koji Mizutani, Christel Lindahl, Stefan Renvert

**Affiliations:** 1 Department of Oral Health, Faculty of Oral Health Science, Kristianstad University, 291 88 Kristianstad, Sweden; 2 Department of Periodontology, Graduate School of Medical and Dental Sciences, Institute of Science Tokyo, Yushima, Bunkyo-Ku, Tokyo Japan; 3 Blekinge Institute of Technology, Karlskrona, Sweden; 4 Division of Restorative Dentistry and Periodontology, Dublin Dental University Hospital, Trinity College Dublin, Dublin, Ireland

**Correction: International Journal of Implant Dentistry (2025) 11:6** 10.1186/s40729-025-00591-0

The original publication of this article contained an incorrect author name. The incorrect and correct information is listed in this correction article, the original article has been updated.

Incorrect:

Viveca Wallin Bentsson

Correct:

Viveca Wallin Bengtsson

